# Caffeine intake reduces sedentary time and increases physical activity predisposition in obese police officers

**DOI:** 10.1590/1414-431X2021e11556

**Published:** 2021-09-20

**Authors:** G.A. Ferreira, L. Wagner, R. Maziero, T. Ataide-Silva, N.A. Silva, R. Bertuzzi, A.E. Lima-Silva

**Affiliations:** 1 Grupo de Pesquisa em Performance Humano, Universidade Tecnológica Federal do Paraná, Curitiba, PR, Brasil; 2 Centro Acadêmico de Vitória, Universidade Federal de Pernambuco, Vitória de Santo Antão, PE, Brasil; 3 Faculdade de Nutrição, Universidade Federal de Alagoas, Maceió, AL, Brasil; 4 Grupo de Estudos em Desempenho Aeróbio, Universidade de São Paulo, São Paulo, SP, Brasil

**Keywords:** Sedentary time, Physical inactivity, Light physical activity, Health, Cardiometabolic risk, Obesity

## Abstract

Sedentary time is associated with increased obesity in police officers. Caffeine intake may reduce sedentary time but it has not been extensively investigated. In the present study, the effect of caffeine ingestion on sedentary time was investigated in obese police officers. Fourteen obese police officers ingested either 5 mg/kg of caffeine or cellulose (placebo) for six days. Information on inactivity time, time spent with physical activities, self-reported perception of tiredness, and physical activity disposition was obtained daily during the intervention period. Sedentary and physical activity times were divided into two intraday periods (T_1_: 08:00 am-02:00 pm and T_2_: 02:00 pm-08:00 pm). Caffeine intake decreased the sedentary time in both T_1_ (79.2±2.2%) and T_2_ (79.1±2.5%), when compared with T_2_ of the placebo condition (81.1±3.6%, P<0.05). Caffeine intake also increased the time spent on light physical activities in T_1_ and T_2_ (17±2 and 18±2%), when compared with T_2_ of the placebo condition (16±3%, P<0.05). In addition, sedentary time increased and light physical activity time decreased from T_1_ to T_2_ in the placebo (P<0.001) but not in the caffeine condition (P=0.81). Caffeine intake had no effect on tiredness (P>0.05), but it increased the self-reported physical activity disposition compared to the placebo condition (4.5±2.7 *vs* 3.2±2.3 units, P<0.05). Caffeine intake reduced the sedentary time and increased the time spent on light physical activities of obese police officers, which seems to be related to a higher disposition for the practice of physical activity.

## Introduction

Sedentary behavior is a multifactorial condition that includes any activity with low energy cost (≤1.5 metabolic equivalent) and it seems to be related to the social development of humanity (e.g., development of technology and occupational tasks) (1). The time spent on such activities is named sedentary time (1). Professional activities in the last years have shifted to longer sitting time, which has increased the sedentary time and led to adverse health outcomes (1).

It has been demonstrated recently that police officers present a higher prevalence of cardiometabolic risk factors such as overweight and obesity than the general population, which seems to be associated with longer sedentary time (2,3). In contrast, a small reduction in sedentary time (∼30 min/day reduction) might help mitigate adverse health outcomes from sedentary occupational tasks (4). For example, some studies have suggested that occasional short bouts of light physical activity performed throughout the day improves aerobic physical fitness and reduces markers of cardiometabolic risk such as waist circumference, postprandial serum glucose and insulin levels (4,5). Therefore, strategies for reducing sedentary time and increasing light- and/or moderate-intensity physical activity might decrease cardiovascular risk factors of police officers that are engaged in sedentary work activities.

Some dietary supplements may change the effects of sedentary time (6). For example, caffeine intake has been shown to increase vigor and reduce tiredness, probably via dopaminergic pathways in the central nervous system (7,8), which might increase the disposition to engage in physical activity (8,9). These psychological alterations induced by caffeine may increase willingness to exercise and break the sedentary lifestyle (10). Thus, caffeine ingestion may be an optimal strategy to reduce sedentary time in obese police officers.

In view of the above, in the present study, we investigated the effects of a 6-day intervention with ingestion of 5 mg/kg body mass of caffeine every morning on sedentary and physical activity times in obese police officers. We also measured the effects of caffeine ingestion on the perception of tiredness and physical activity predisposition. Our hypotheses were: 1) caffeine ingestion reduces tiredness and increases the predisposition for physical activity, which in turn reduce sedentary time and increase physical activity.

## Material and Methods

### Participants

Fourteen obese (body mass index ≥30 kg/m^2^) men with (means±SD) 41.0±4.0 years of age, height of 173±5 cm, body mass of 101.8±11.4 kg, abdominal circumference of 107.1±7.0 cm, waist circumference of 105.3±6.9 cm, body mass index of 33.9±3.1 kg/m^2^, and fat mass of 33.4±1.2% were enrolled in this study. Participants were police officers from the Preventive Police of the State of Parana, Brazil. The inclusion criteria for participation in this study were: 1) age between 30 and 50 years; 2) body mass index higher than 30 kg/m^2^; 3) non-smoker; and 4) not using medications that affect energy intake and expenditure. Participants signed a written informed consent form after being informed of experimental procedures and possible risks. This study was approved by the Human Ethics Committee of the Federal University of Technology of Paraná.

### Study design

Participants took part in two 6-day intervention periods, with a 1-week washout period between the two interventions. One intervention period consisted of six consecutive days (from Thursday to Tuesday) of taking the same substance every day (caffeine or placebo). The two interventions were performed using a counterbalanced, crossover, and placebo-controlled design. Twenty-four hours prior to each intervention period, anthropometric assessments were performed, and participants were guided on how to report dietary records and perception of tiredness and physical activity predisposition. Participants also received an accelerometer and were instructed on how to use it during the intervention period. Twenty-four hours after the last day of each intervention period, anthropometric measurements were retaken.

### Supplementation schedule

Participants received six capsules containing either 5 mg/kg body mass of caffeine or 5 mg/kg body mass of cellulose (placebo). Participants took one capsule of the same substance (caffeine or placebo) each morning (08:00 am), for six consecutive days, and they were blinded about the substance. Participants were instructed to eat and drink as usual during the intervention period. Participants were also allowed to consume caffeinated beverages and foods during the intervention.

### Measurements

#### Sedentary and physical activity times

Sedentary and physical activity times were monitored using an ActiGraph wGT3X-BT accelerometer (ActiGraph, USA). Participants were instructed to wear the accelerometer on their wrist for the entire day (from waking until bedtime) for six consecutive days of each intervention. Data were considered valid when the accelerometer recorded more than 10 h of continuous activity, as previously suggested (5). Non-wear time was defined as 60 min or more with zero counts (5). Data from the accelerometer were analyzed in two intraday windows: 1) from 08:00 am to 02:00 pm (T_1_) and 2) from 02:00 pm to 08:00 pm (T_2_). The analysis using intraday windows was chosen to match T_1_ with the caffeine plasma half-life (∼6 h) (11). The time of sedentary behavior (<100 counts/min), and of light (from 100 to 1,951 counts/min), moderate (from 1,952 to 5,724 counts/min), and vigorous (≥5,725 counts/min) physical activity were calculated (12).

#### Self-reported tiredness and physical activity predisposition

The perception of tiredness and physical activity predisposition were measured every day at 08:00 pm during the intervention period using a 10-point categorical scale, which is a valid and practical tool to assess the intensity of a subjective psychophysical feeling (13). The categorical scale ranged from zero (nothing at all) to 10 (extremely). For the following questions, participants assigned a number corresponding to their perceived sensation (13): 1) Tiredness: “*How tired were you when performing your life activities today?*” and 2) Physical activity predisposition: “*How disposed were you to perform physical activity today?*”.

#### Anthropometric assessments

Height, body mass, and abdominal and waist circumferences were measured at 11:00 am on the day before and the day after the intervention. All measurements were taken by the same experienced evaluator, who was blinded to which condition participants were allocated. Height was measured to the nearest 0.1 cm using a portable stadiometer (Sanny, Brazil) and body mass to the nearest 0.1 kg using an electronic scale (Filizola, Brazil). Body mass index was calculated by dividing body mass (kg) by height squared (m^2^). Waist and abdominal circumferences were obtained to the nearest 0.1 cm using a non-stretchable metric tape measure (Sanny). Waist circumference was measured at the smallest perimeter between the iliac crest and the last rib. Abdominal circumference was measured at two anatomical points: 1) at the half distance between the xiphoid process and umbilical scar and 2) at the umbilical scar (14). The mean between these two anatomical points was used to estimate percentage of fat mass using the following equation recommended for obese men (14): %Fat mass = 0.31457 (AB) - 0.10969 (BM) + 10.8336, where AB is the mean between the two abdominal circumferences in cm and BM is body mass in kg.

#### Food consumption

Participants filled out a dietary record during the six days of each intervention. The dietary records were analyzed for total daily energy, macronutrients, and caffeine intake using a nutrition software (Nutrition Support Software, NutWin^®^ software, UNIFESP, Brazil). Data from food and caffeine intake were separately reported for T_1_ and T_2_ periods (Supplementary Table S1).

### Statistical analysis

Data distribution was verified using the Shapiro-Wilk test. Three-way repeated-measures analysis of variance (ANOVA) was used to determine the effect of substance (placebo and caffeine), period (T_1_and T_2_), and day (from Thursday to Tuesday) for sedentary time and physical activity time. Two-way repeated-measures ANOVA was used to determine the effect of substance (placebo and caffeine) and day (from Thursday to Tuesday) on self-reported tiredness and physical activity predisposition. Two-way repeated-measures ANOVA was also used to determine the effect of substance (placebo and caffeine) and time (pre- and post-intervention) for anthropometric variables. Fisher's least significant difference was used as the *post hoc* test to determine the differences detected by ANOVA. The level of significance was set at P<0.05. Statistical analyses were performed using Statistic software, version 10 (StataSoft, Inc.^®^, USA).

## Results

### Sedentary time and physical activity time

#### Sedentary time

Three-way ANOVA showed a condition-period interaction for sedentary time (F_(1,13)_=11.8, P=0.004, Supplementary Table S2). Caffeine intake decreased sedentary time in T_1_ (79.2±2.2%) and T_2_ (79.1±2.5%) compared with T_2_ of the placebo (81.1±1.8%, P=0.003 and P=0.002) but not T_1_ of the placebo (79.7±2.3%, P=0.39 and P=0.53). Sedentary time in the caffeine condition did not differ between T_1_ and T_2_ (P=0.81), but sedentary time was higher in the T_2_ period compared with the T_1_ period for the placebo condition (P<0.001). There was also a main effect of day (F_(5,65)_=3.0, P=0.02), in which sedentary time was higher on Sunday than on other days (all P<0.006, Supplementary Table S2).

#### Light physical activity time

Three-way ANOVA showed a condition-period interaction (F_(1,13)_=11.5, P=0.005, Supplementary Table S2) for light physical activity time. Caffeine intake increased light physical activity time in T_1_ (17.8±1.9%) and T_2_ (18.7± 2.3%) compared with T_2_ of the placebo (16.3±2.8%, P=0.02 and P=0.001) but not with T_1_ of the placebo (18.1±1.8%, P=0.79 and P=0.15). Light physical activity time in the caffeine condition did not differ between T_1_ and T_2_ (P=0.10), but light physical activity was lower in the T_2_ period compared with the T_1_ period for the placebo condition (P=0.01). There was also a main effect of day (F_(5,65)_=3.0, P=0.02), in which light physical activity time was lower on Sunday than on other days (all P<0.05, Supplementary Table S2).

#### Moderate and vigorous physical activity times

There was only a main effect of day for moderate physical activity (F_(5,65)_=6.2, P<0.001, Supplementary Table 2), with less time spent on moderate physical activity on Sunday than on other days (P<0.05). No time was spent on vigorous physical activity in any condition.

### Self-reported physical activity predisposition and tiredness

**Figure 1:**
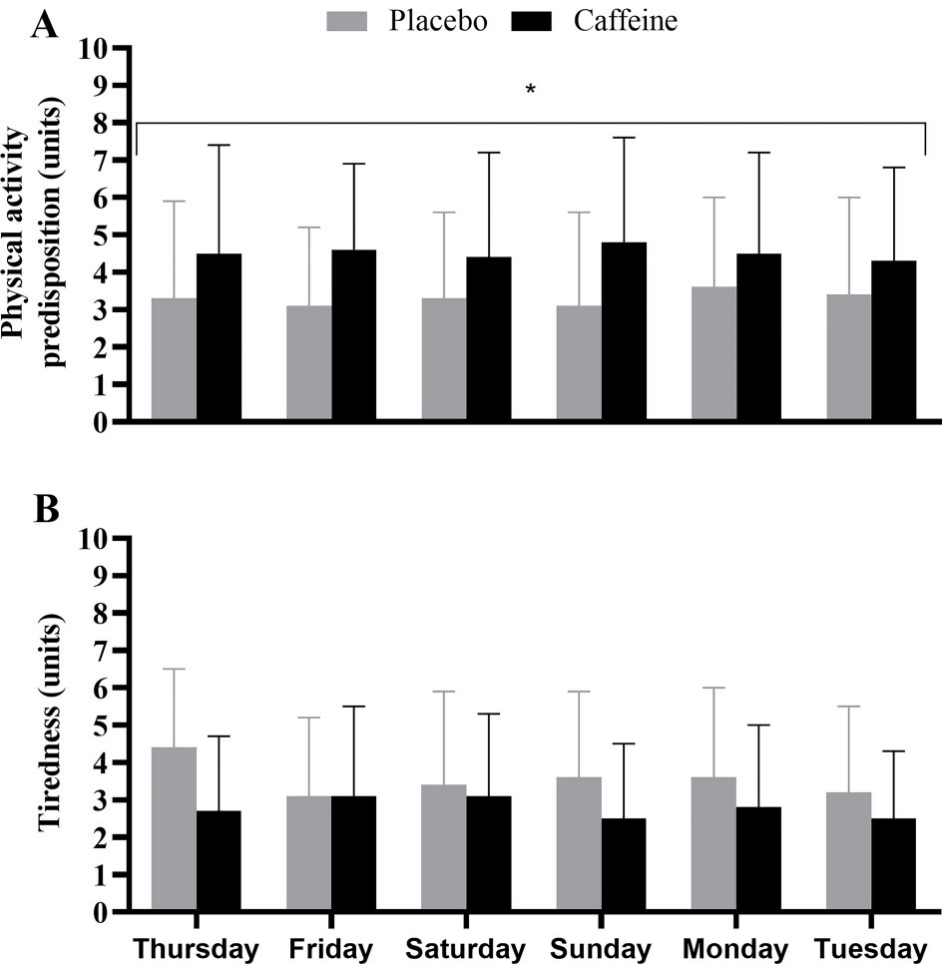
Self-reported physical activity predisposition (**A**) and tiredness (**B**). Data are reported as means±SD for n=14. *P<0.05 caffeine condition compared to the placebo condition (repeated measures ANOVA).

Self-reported physical activity predisposition and tiredness are shown in Figure 1. Caffeine intake increased the predisposition for physical activity (main effect of condition, F_(1,12)_=5.0, P=0.046), but had no effect on tiredness (F_(1,12)_=1.3, P=0.27). There were no other main effects or interactions (all P>0.05).

### Anthropometric measurements

Anthropometric measurements were similar between placebo and caffeine conditions and did not change from pre- to post-intervention (all P>0.05, Table 1).

**Table 1:** Anthropometric measurements pre- and post-six days of daily placebo or caffeine ingestion.

	Placebo		Caffeine	
	Pre	Post	Pre	Post
Body mass (kg)	102.1±11.5	102.0±11.1	102.1±11.5	101.9±11.4
Body mass index (kg/m^2^)	34.0±3.2	33.9±3.1	34.0±3.0	33.9±3.1
Body fat (%)	33.5±1.2	33.8±0.6	33.7±0.9	33.8±0.9
Abdominal circumference 1 (cm)	105.3±6.4	107.4±6.3	105.8±5.8	107.1±5.1
Abdominal circumference 2 (cm)	109.8±7.7	109.7±7.5	110.5±7.0	109.9±7.0
Waist circumference (cm)	105.2±7.2	105.2±7.3	104.8±6.0	104.5±6.3

Abdominal circumference 1 was measured at half distance between the xiphoid process and umbilical scar. Abdominal circumference 2 was measured at the umbilical scar. Data are reported as means±SD, for n=14. There were no significant differences between conditions or between pre- and post-intervention (P>0.05, ANOVA).

### Food consumption

The total energy and macronutrient intake remained stable throughout the period and was similar between placebo and caffeine conditions (all P>0.05, Supplementary Table S1). The diet consisted of 49±17% carbohydrates, 31±13% fats, and 20±11% proteins for the placebo condition, and 47±18% carbohydrates, 31±12% fats, and 22±14% proteins for the caffeine condition. Caffeine supplementation had no effect on daily caffeine intake (P>0.05), which varied slightly throughout the intervention (P<0.05). The total daily caffeine intake was ∼1.4 mg/kg body mass.

## Discussion

Consistent with our first hypothesis, caffeine increased predisposition for physical activity, resulting in reduced sedentary time and increased light physical activity time in obese police officers but without affecting tiredness.

Participants of the present study spent ∼24 min/day in moderate physical activity, close to the limit of recommended physical activity guidelines (i.e., at least 150 min of moderate physical activity per week (15)). It is noteworthy, however, that police officers spent the majority of their time performing sedentary activities (i.e., ∼80% of total time in sedentary behavior). Sedentary time is associated with a higher body mass index and waist circumference (5) and several other markers of cardiometabolic risk (4,5). Adverse health outcomes in police officers are directly related to a high amount of time spent in sedentary activities during their work (2,3). Thus, the challenge is to promote lifestyle changes in these workers to reduce their sedentary time and increase their light to moderate physical activities (15).

In the present study, caffeine intake reduced sedentary time and increased light physical activity time. To our knowledge, only one previous study investigated the effects of caffeine intake on sedentary behavior (6). That study recruited lean men (BMI <25 kg/m) and failed to find any effect of four days of caffeine ingestion (two daily doses of 2.5 mg/kg body mass) on sedentary behavior (6). Participants were physically active (∼49 min of daily moderate physical activity) and sedentary time was ∼700 min per day (6). Some methodological differences may explain the different outcomes between our results and those of that study (6). First, we did not restrict usual caffeine intake (∼1.4 mg/kg body mass) to maximize the practical significance of the results, since from a practical perspective, caffeine supplementation could be performed without suspending the ingestion caffeinated beverages and food. Second, we recruited obese individuals with a low daily physical activity time (∼24 min of daily moderate physical activity) and greater sedentary time (∼1210 min of daily). It has been suggested that a slight reduction in sedentary behavior of ∼30 min/day may be sufficient to decrease body mass and the body mass index (4). In the present study, caffeine intake reduced sedentary time by 2% (∼24 min/day), which was replaced by time spent in light physical activity (∼15 min/day). Together, these findings suggested that caffeine intake might be effective in changing sedentary behavior, which seems to be related with increase in light physical activity time in obese police officers.

Another important finding of the present study was that sedentary time increased, while light physical activity time decreased from morning to afternoon in the placebo but not in the caffeine condition. An increase in sedentary time from morning to evening has been reported in the literature (16), but no previous study has investigated the potential of caffeine ingestion to contain the increase in sedentary time during the day. Our findings indicated that caffeine prevented the increase in sedentary time in the afternoon in police officers. Interestingly, caffeine also increased self-reported physical activity predisposition, which may have favored an increase in light physical activity time in the afternoon. Higher levels of physical activity have been negatively associated with fatigue/weakness (10). In addition, caffeine ingestion did not affect tiredness, which suggested that caffeine may alter physical activity predisposition/tiredness ratio. This may indicate that the likelihood of performing physical activity increases with a lower tiredness perception. It has been suggested that caffeine intake affects mood, especially self-perceived vigor, increasing the predisposition to physical activity and exercise (8). Caffeine intake makes exercising more pleasant and less difficult than without caffeine intake (17). Our findings also showed that an increased physical activity predisposition with caffeine intake may counteract the accumulation of fatigue and exhaustion during the day, which in turn helps to prevent the natural tendency to increase sedentary behavior in the afternoon.

An acute reduction in sedentary time caused by caffeine ingestion has the potential to reduce cardiometabolic risk (4,5). Although the effects of chronic caffeine intake on cardiometabolic risk have not been investigated, studies have demonstrated that increased consumption of food containing caffeine reduces cardiometabolic risk (18). Specifically, coffee intake is associated with low cardiometabolic risks such as obesity, metabolic syndrome, and type 2 diabetes (19), but direct comparison between caffeine and coffee intake is difficult because coffee contains other substances that may reduce energy intake. In the present study, 6 days of increased caffeine intake was not sufficient to reduce some markers of cardiometabolic risk (e.g., body fat and abdominal and waist circumferences). Thus, the effect of long-term caffeine ingestion on cardiometabolic risk warrants further research. It is noteworthy, however, that caffeine intake decreased sedentary behavior and increased light physical activity by an amount (∼24 and 16 min/day, respectively) considered sufficient to reduce other non-measured markers of cardiometabolic risk (4,5). Further studies measuring other markers of cardiometabolic risk are necessary to explore the potential of caffeine intake. Finally, volunteers continued having their usual caffeinated beverages and foods; therefore, a summation effect of supplemented and food- and beverage-derived caffeine intake on our main outcomes cannot be excluded. However, there was no difference between dietary caffeine intake between placebo and caffeine conditions, which may argue against a potential interaction between supplemented and food- and beverage-derived caffeine intake. While not eliminating the habitual caffeine intake could be criticized, it should be emphasized that this approach is clinically relevant as it offers insight into the functional impact of a new intervention.

Some limitations should be highlighted. We did not assess the effectiveness of blinding, which is important in studies with subjective outcomes (20). Nevertheless, to minimize a potential risk of bias from inadequate blinding, we used a double-blind design. Furthermore, we did not assess the pre-intervention physical activity levels of participants, but the accelerometer data analysis in the placebo condition indicated that participants were inactive. Although the placebo may affect psychological factors (20) that could influence physical activity, the randomized study design might have mitigated this influence.

In conclusion, short-term caffeine intake reduced sedentary time and increased light physical activity, which might be related to a caffeine-induced increase in physical activity predisposition.

## Supplementary Material

Click here to view [pdf].
